# Supplementary material for: WordCommentsAnalyzer: A windows software tool for qualitative research
Source: F1000Res. 2018 Sep 4;7:536. Originally published 2018 May 3. [Version 2] doi: 10.12688/f1000research.14819.2 (PMC6137411; doi:10.12688/f1000research.14819.2)
Supplement: Supplementary file 3 [file f1000research-7-17614-s0002.tgz › b2a79496-4ada-47c4-9682-a99ce867836d.zip!/Eight Iranian Health Journals/Salmand_Iranian Journal of Ageing _12.docx]

© Salmand_Iranian Journal of Ageing . This work is licensed under CC BY-NC 4.0 (https://creativecommons.org/licenses/by-nc/4.0/).

Volume & Issue: دوره 10 - شماره 2

Title: یکپارچگی اجتماعی و کیفیت زندگی مرتبط با سلامت سالمندان مراجعه‌کننده به مراکز درمانی شهرستان سبزوار در سال 1390

http://salmandj.uswr.ac.ir/article-1-730-fa.html

اهداف سالمندی دوره‌ای از زندگی است که عوامل بسیاری،کیفیت زندگی سالمندان را تحت تاثیر قرار می‌دهد. از جمله موارد موثر بر کیفیت زندگی و سلامت سالمندان، تعاملات اجتماعی است. مطالعه حاضر با هدف بررسی رابطه یکپارچگی اجتماعی و کیفیت زندگی مرتبط با سلامت سالمندان شهرستان سبزوار، انجام شد.  مواد و روش ها این مطالعه‌ی توصیفی-تحلیلی بر روی 135 سالمند مراجعه‌کننده به دو مرکز اصلی درمانی شهرستان سبزوار (واسعی- امداد) به روش نمونه‌گیری طبقه‌ای متناسب با حجم، انجام شد. ابزارهای پژوهش شامل پرسشنامه کیفیت زندگی مرتبط با سلامت SF-36، پرسش‌نامه محقق ساخته یکپارچگی اجتماعی و فرم اطلاعات دموگرافیک بود. اطلاعات کسب شده با استفاده از روش‌های آماری توصیفی و همبستگی پیرسون و اسپیرمن، آزمون تی، من ویتنی و آنالیز واریانس، مورد تجزیه و تحلیل قرار گرفتند. یافته ها میانگین نمره یکپارچگی اجتماعی در کل سالمندان 5/9±6/30 و در سطح متوسط بود و در مردان و زنان سالمند به ترتیب 4/9±26/33 و 6/9±46/27 بود. نمره کیفیت زندگی مرتبط با سلامت در بعد سلامت جسمی 65/2±87/10 و در بعد سلامت روانی 96/2±14/11 بود. بین یکپارچگی اجتماعی و کیفیت زندگی مرتبط با سلامت، همبستگی مستقیم مثبت و معناداری وجود داشت (001/0>P، 73/0r₌). بین سن (001/0=P، 3/r₌0-)، وضعیت تاهل (001/0>P)، اشتغال(001/0>P)، جنسیت (001/0>P) و سطح تحصیلات (003/0=P، 4/0r₌) با یکپارچگی اجتماعی، رابطه معناداری وجود داشت. نتیجه گیری با توجه به رابطه بین یکپارچگی اجتماعی با ابعاد کیفیت زندگی مرتبط با سلامت سالمندان، سیاست‌گذاران حوزه سلامت، می‌توانند با انجام مداخلاتی در زمینه یکپارچگی اجتماعی، موجب ارتقای کیفیت زندگی سالمندان شوند.

Title: بررسی چگونگی ارتقای فعالیت‌های خارج از منزل سالمندان از طریق طراحی محصولات (طراحی کیف خرید) کاربر‌محور: یک مطالعه کیفی به روش کاربرمحور

http://salmandj.uswr.ac.ir/article-1-863-fa.html

اهداف افراد در دوران سالمندی با محدودیت‌های فیزیکی مختلفی روبه‌رو می‌شوند و این امر نه تنها فعال ماندن به لحاظ فردی و اجتماعی را دشوار می‌کند بلکه بر سلامت روانی سالمندان نیز اثرگذار است. طراحی (طراحی محصولات، فضا و غیره) و ارائه راه‌حل‌های نوین در جهت تسهیل زندگی سالمندان، می‌تواند منجر به افزایش سطح فعالیت‌های فردی و اجتماعی آنان شود. مواد و روش ها مقاله حاضر بخشی از مطالعه‌ای کیفی با رویکرد کاربر‌محور است که به بررسی فعالیت‌های اجتماعی خارج از منزل سالمندان و پیشنهاد محصولی در جهت ارتقا آن ‌می‌پردازد. داده‌های این مطالعه با روش نمونه‌گیری تصادفی و از طریق پخش پرسش‌نامه، مشاهده، مصاحبه و ثبت بازخوردهای شرکت‌کنندگان به دست آمده است. مطالعه‌ کیفی حاضر به ترتیب در چهار مرحله انجام شده است: 1.پخش پرسش‌نامه بین 42 نفر سالمند 2. مصاحبه با 19 نفر سالمند 3. ثبت بازخورد و نظرات 19 نفر سالمند 4. پخش پرسش‌نامه رضایتمندی بین 19 نفر سالمند شرکت‌کننده در مراحل آخر جهت سنجش رضایتمندی از طرح پیشنهادی محصول. تعداد 12 نفر سالمند زن و 7 نفر سالمند مرد در سه مرحله آخر مشارکت کرده و داده‌ها بر اساس نظرات ایشان مطالعه و بررسی و نمونه‌های مشابه و مرتبط با محصول، جمع‌آوری شد و سپس بر اساس تحلیل داده‌ها، راهکارها ارائه شد. یافته ها در مرحله اول اصلی‌ترین فعالیت خارج از منزل، خرید و پر‌رفت و آمدترین مراکز خرید،  مراکز میوه و تره‌بار شناسایی شد. کیف خرید به عنوان محصول مرتبط با پروسه خرید انتخاب شد و  سپس از طریق مصاحبه به شناخت مشکلات آن، پرداخته شد. در طراحی محصول نهایی به قابلیت تنظیم، ایستایی و ایمن بودن محصول که حس رضایتمندی را بالا ببرد و حس وابستگی و نیاز به دیگران را کاهش دهد، توجه شد.  نتیجه گیری در طراحی محصول نهایی به  مشکلات مطرح شده توسط سالمندان، توجه شد. برای پژوهش‌های احتمالی آینده در نظر گرفتن اندازه‌های بدنی افراد به منظور تطابق راه‌حل‌های احتمالی با نیازهای خاص هر فرد، پیشنهاد می‌شود. از سویی دیگر بررسی عادات و رفتارهایی که موجب صدمه به سالمند در پروسه خرید می‌شود و تعدیل آن‌ها از طریق ارائه راه‌حل‌های مناسب در طراحی محصول، می‌تواند در ارتقای کیفیت فعالیت‌های این گروه بسیار موثر باشد.

Title: بررسی تأثیر تفریح درمانی بر میزان افسردگی در سالمندان مراجعه‌کننده به مراکز مراقبت روزانه منتخب شهر تهران

http://salmandj.uswr.ac.ir/article-1-821-fa.html

اهداف امروزه با افزایش تعداد سالمندان، توجه به مشکلات ایشان در جامعه، ابعاد تازه و گسترده‌ای پیدا کرده است. بنا به آمار، یکی از مهم‌ترین مشکلات روان‌شناختی این قشر، افسردگی است. در این راستا این مطالعه با هدف بررسی تأثیر تفریح درمانی بر میزان افسردگی در سالمندان انجام پذیرفت. مواد و روش ها پژوهش حاضر یک تحقیق نیمه‌تجربی است. جامعه پژوهش شامل کلیه‌‌ی سالمندان بالای 60 سال واجد شرایط معیار ورود و مراجعه‌کننده به مرکز مراقبت روزانه‌ی یاس شهر تهران بود (002n=). 64 نفر بر اساس حجم نمونه‌ی محاسبه شده به صورت تصادفی (به کمک جدول اعداد تصادفی) انتخاب شدند و با استفاده از گزینش تصادفی (شیر-خط) در دو گروه آزمون (32 نفر) و شاهد (32 نفر) قرار گرفتند؛ دو نفر از گروه شاهد به علت عدم مراجعه، حذف شدند. مداخله، شامل10جلسه تفریح درمانی در طول10هفته برای گروه آزمون بود. افسردگی قبل و بعد از مداخله و همچنین دو هفته بعد از مداخله در هر دو گروه اندازه‌گیری شد. ابزار پژوهش شامل پرسشنامه‌های GDS (آلفا: 9/0)، AMT (آلفا: 99/0) و Barthel (آلفا: 93/0) بود. برای تجزیه و تحلیل داده‌ها از نرم‌افزار SPSS نسخه 18 استفاده شد. تجزیه و تحلیل داده‌ها به کمک آزمون‌ آماری کای اسکوئر، آزمون تی مستقل، من ویتنی، مدل معادله‌ی برآورد تعمیم یافته و آزمون شاپیرو-ویلک صورت پذیرفت.  یافته ها یافته‌های مطالعه نشان داد که در گروه آزمون، میانگین نمرات افسردگی، قبل، بعد و در پیگیری دو هفته بعد از انجام مداخله به ترتیب2/4±78/5، 12/2±59/2، 2/2±03/3 و در گروه شاهد به ترتیب 01/3±20/5، 9/2±16/5 و 8/2±5 بود. علاوه بر این یافته‌های مطالعه نشان داد که قبل از انجام مداخله، میانگین افسردگی در دو گروه آزمون و شاهد دارای اختلاف معنی‌داری نبود (932/0P=)، اما بعد از انجام مداخله و در پیگیری دو هفته بعد از آن اختلاف معنی‌دار بود ( به ترتیب 001/0=P و 007/0P=). از طرف دیگر معادله برآورد تعمیم یافته در گروه آزمون نشان داد که اختلاف نمرات افسردگی در مراحل پیش آزمون، پس آزمون و پیگیری در گروه آزمون معنی‌دار است (0001/0P<)، در حالی که در گروه شاهد تفاوت معنی‌دار نیست (298/0P=).  نتیجه گیری یافته‌ها نشان داد که تفریح درمانی، موجب کاهش معنادار افسردگی سالمندان شده است.

Title: مقایسه سلامت روانی مردان سالمند در سه گروه ورزشکار و فعال و غیرفعال

http://salmandj.uswr.ac.ir/article-1-636-fa.html

اهداف هدف پژوهش حاضر مقایسه سلامت روانی و خرده مقیاس‌های آن بین مردان سالمند ورزشکار و فعال و غیرفعال بود. بدین منظور 150 نفر از مردان سالمند در قالب سه گروه ورزشکار، فعال و غیرفعال انتخاب و اقدام به تکمیل پرسشنامه‌های مربوطه نمودند. مواد و روش ها  این پژوهش از نوع همبستگی است. برای سنجش سلامت روان آزمودنی‌ها از پرسشنامه سلامت روانی 28-GHQ استفاده شد. همچنین برای گمارش افراد سالمند به دو گروه فعال و غیر‌فعال از دو پرسشنامه‌ی فعالیت جسمانی یال و پرسشنامه‌ی میزان فعالیت بدنی بک استفاده شد. گروه ورزشکار از بازیکنان بالای60 سال تیم‌های پیشکسوتان رشته ورزشی فوتبال شهر تهران انتخاب شدند. برای مقایسه میانگین گروه‌ها از تحلیل واریانس یک راهه استفاده شد.  یافته ها نتایج تحقیق نشان داد که سلامت روانی و دو خرده‌مقیاس اضطراب و کارکرد اجتماعی و همچنین میزان فعالیت بدنی هر سه گروه نسبت به یکدیگر تفاوت معنی‌داری داشت (01/0>P). دو خرده‌مقیاس علائم جسمانی و افسردگی بین گروه فعال و گروه ورزشکار با گروه غیرفعال تفاوت معنی‌داری را نشان داد (01/0P<) ولی بین گروه ورزشکار و فعال، اختلاف مذکور، معنی‌دار نبود. نتیجه گیری با توجه به نتایج حاصل از این تحقیق می‌توان نتیجه گرفت که برنامه‌های حرکتی و ورزشی متناسب با ویژگی‌های ساختاری و روانشناختی سالمندان، علاوه بر تأمین سلامت جسمانی، فرد سالمند را از لحاظ روانی برای زندگی هرچه بهتر و سالم‌تر آماده می‌کند.

Title: رابطه سواد سلامت و وضعیت سلامت جسمانی در سالمندان شهر کرمان

http://salmandj.uswr.ac.ir/article-1-790-fa.html

اهداف سالمندان به عنوان یکی از مهم‌ترین گروه‌های در معرض خطر اثرات ناخوشایند سطح پایین سواد سلامت به شمار می‌آیند. این مطالعه با هدف تعیین ارتباط سواد سلامت و وضعیت سلامت در سالمندان شهر کرمان انجام شد. مواد و روش ها مطالعه‌ حاضر یک مطالعه مقطعی است که در آن 200 نفر از سالمندان شهر کرمان مورد پرسشگری قرار گرفتند. به منظور جمع‌آوری اطلاعات در خصوص سواد سلامت از پرسش‌نامه سواد سلامت عملکردی بزرگسالان و برای اندازه‌گیری سلامت جسمانی از ابزار سنجش توانائی در انجام فعالیت روزمره سالمند و فرم کوتاه شده‌ی 10 سوالی پرسشنامه 36-SF استفاده شد. برای آنالیز داده‌ها از شاخص‌های توصیفی و نیز برای بررسی ارتباط متغیرها از آزمون آماری کای دو،t  و آنالیز واریانس یک طرفه استفاده شد. یافته ها بیشتر سالمندان مورد مطالعه از سطح سواد سلامت ناکافی برخوردار بودند (%5/52). بین سطح سواد سلامت با تحصیلات (P=0/021) و شغل(P=0/041) رابطه معنی‌داری مشاهده شد. همچنین رابطه‌ بین سواد سلامت و توانائی انجام فعالیت‌های روزانه معنی‌دار بود (P=0/038). بین سواد سلامت با سن، جنسیت، وضعیت تأهل، وضعیت مالی و عملکرد فیزیکی رابطه آماری معنی‌دار مشاهده نشد. نتیجه گیری بهبود سواد سلامت ممکن است استراتژی موثری در بهبود وضعیت سلامت سالمندان باشد. با توجه به نتایج پژوهش، آموزش و توسعه‌ی رفتارهای ارتقا‌دهنده سلامت در سالمندان و لزوم توجه بیشتر به امر سواد سلامت در برنامه‌های ارتقای سلامت توصیه می‌شود.

Title: تأثیر تمرینات ثبات‌دهنده مرکزی بر ترکیب بدنی و چربی خون زنان یائسه سالمند

http://salmandj.uswr.ac.ir/article-1-774-fa.html

اهداف کاهش سطح استروژن پس از یائسگی با تغییرات نامطلوب ترکیب بدنی و چربی خون ارتباط دارد. در این پژوهش اثر 24 هفته تمرین قدرتی ثبات ‌دهنده مرکزی بر ترکیب بدنی و چربی خون زنان یائسه‌ی سالمند بررسی شد. مواد و روش ها این تحقیق، یک مطالعه نیمه تجربی است که در سال 1391 انجام شد. 23 زن یائسه سالمند مقیم آسایشگاه سالمندان شهر شاهرود به‌طور هدفمند انتخاب و به طور تصادفی در دو گروه تجربی و کنترل قرار گرفتند. برنامه‌ی تمرینی به مدت 24 هفته، هفته‌ای 3 روز و هر روز 45 تا 60 دقیقه زیر نظر مربی انجام شد. 30-25 دقیقه از هر جلسه تمرین به تمرینات ثبات مرکزی (قدرت و استقامت) اختصاص داده شد. چربی خون و شاخص‌های آنتروپومتریکی چاقی (وزن بدن، شاخص توده بدن، اندازه دور کمر)، قبل و بعد از برنامه‌ی تمرین اندازه‌گیری شد.   یافته ها نتیجه آزمون t همبسته نشان داد بین اندازه دور کمر گروه تجربی قبل و بعد از تمرین اختلاف معناداری وجود داشت (03/0=P). نتیجه آزمون t مستقل نیز نشان داد فقط بین سطح کلسترول تام گروه تجربی و کنترل پس از تمرین اختلاف معناداری وجود داشت (007/0=P) و تغییرات در سایر اجزای چربی خون معنادار نبود. نتیجه گیری براساس یافته‌های پژوهش حاضر، تمرینات ثبات‌دهنده مرکزی که شامل تمرینات قدرتی و استقامتی است، می‌تواند باعث بهبود ترکیب بدنی و برخی اجزای چربی خون زنان یائسه سالمند شود.

Title: اثربخشی آموزش گروهی توکل به خدا بر افسردگی سالمندان مرد مقیم آسایشکاه کهریزک

http://salmandj.uswr.ac.ir/article-1-677-fa.html

اهداف هدف از انجام این پژوهش، بررسی اثربخشی آموزش توکل به خدا بر کاهش افسردگی سالمندان مرد بود. مواد و روش ها پژوهش حاضر ازنوع مطالعات نیمه‌آزمایشی با طرح پیش‌آزمون و پس آزمون با گروه کنترل بود. جامعه آماری شامل سالمندان مرد ساکن در آسایشگاه خیریه سالمندان کهریزک بود که به صورت تصادفی از بین آسایشگاهای استان تهران انتخاب شدند. فرم 15 سوالی GDS بر روی 50 نفر از سالمندان اجرا شد و 20 نفر که نمرات افسردگی‌شان در مقیاس مذکور، 8 یا بالاتر از 8 بود، انتخاب و به صورت تصادفی به دو گروه 10 نفری آزمایش و گواه تقسیم  شدند. در این پژوهش برای گروه آزمایش، 8 جلسه‌ آموزش توکل به خدا برگزار شد. یک هفته پس از اتمام آموزش گروهی، پس آزمون افسردگی GDS شامل 15سوال بر روی هر دو گروه آزمایش و گواه اجرا شد و نتایج با استفاده از آزمون تحلیل کوواریانس یک‌راهه (آنکوا) مورد بررسی قرار گرفت.  یافته ها برای تحلیل داده‌ها از آزمون تحلیل کوواریانس که بیانگر معنادار بودن مداخله آموزشی بر آزمودنی‌ها است، استفاده شد (P<0/05). نتیجه گیری بر اساس یافته‌های پژوهش، باور دینی توکل به خدا بر کاهش افسردگی سالمندان موثر بوده است.

Title: رابطه حمایت اجتماعی و رضایت از زندگی با شادکامی در سالمندان ساکن منازل شخصی شهر شیراز

http://salmandj.uswr.ac.ir/article-1-683-fa.html

اهداف هدف از این پژوهش بررسی رابطه‎ی حمایت اجتماعی و رضایت از زندگی با شادکامی در سالمندان ساکن منازل شخصی شهر شیراز بود. مواد و روش ها نوع تحقیق کاربردی و روش پژوهش همبستگی است. جامعه آماری شامل کلیه‌ی سالمندان زن و مرد ساکن در خانه‌های شخصی شهر شیراز بود. با روش نمونه‌گیری در دسترس، نمونه‌ای با حجم 379 نفر انتخاب شد. داده‌ها از طریق پرسشنامه‌های حمایت اجتماعی فیلیپس، رضایت از زندگی داینر و شادکامی و افسردگی ژوزف و لوئیس جمع‌آوری شد. یافته ها نتایج تحلیل داده‌ها، همبستگی مثبت و معنادار بین مولفه‌های حمایت اجتماعی و رضایت از زندگی با شادکامی را نشان داد. از بین مولفه‌های حمایت اجتماعی، حمایت خانواده و دوستان و همچنین رضایت از زندگی، شادکامی را به صورت مثبت و معنی‌دار پیش‌بینی کرد.  نتیجه گیری بالا بودن حمایت اجتماعی و رضایت از زندگی در سالمندان می‌تواند باعث افزایش شادکامی در این افراد شود. میزان حمایت‌هایی که سالمندان از خانواده و دوستان خود دریافت می‌کنند و همچنین میزان رضایت از زندگی،  می تواند میزان شادکامی آن ها را پیش‌بینی کند.

Title: بررسی طول مدت بستری غیر ضروری و هزینه‌های آن در سالمندان مبتلا به سکته مغزی در بخش مراقبت حاد بیمارستان شهید دکتر فیاض بخش تهران در سال1392

http://salmandj.uswr.ac.ir/article-1-819-fa.html

اهداف هزینه‌های نظام سلامت به دلایل متعددی ازجمله افزایش جمعیت سالمندان به طور فزآینده‌ای رو به افزایش است. استفاده بهینه ازمنابع محدود مالی و انسانی به ویژه در بخش بستری که سهم قابل توجهی از هزینه‌های نظام سلامت را به خود اختصاص می‌دهد، بیش از پیش ضروری است. هدف از انجام  این مطالعه، بررسی طول مدت بستری غیر ضرور و هزینه‌های آن در سالمندان مبتلا به سکته مغزی در بخش مراقبت حاد بیمارستان شهید دکتر فیاض بخش تهران در سال1392 بود. مواد و روش ها این پژوهش یک مطالعه توصیفی است که از سالمندان مبتلا به سکته مغزی بستری شده در بخش داخلی اعصاب (مراقبت حاد) بیمارستان شهید دکتر فیاض بخش تهران به عمل آمده است. یافته ها به طور متوسط هر بیمار مبتلا به سکته مغزی 3/8 روز در بیمارستان بستری شده است. در حدود 59% از این بیماران می‌توانستند بخشی از خدمات بستری خود را طبق نظر متخصصین در بخش تحت حاد دریافت نمایند. به عبارت دیگر با انتقال مدتی از اقامت بیمار به بخش تحت حاد، مدت زمان بستری بیماران، قابل کاهش به 2/6 روز است. ضمنا میانگین هزینه هر بیمار و هر روز بستری دروضعیت موجود به ترتیب برابر با 000/ 800/11و 000/ 416/1 ریال بوده است که در صورت میسر بودن ادامه درمان بیماران در بخش تحت حاد، این رقم حدود 17% قابل کاهش بود.  نتیجه گیری نتایج این پژوهش نشان داد که %25 از روز‌های بستری و %17 از هزینه‌هایی که در بخش  مراقبت حاد اتفاق می‌افتد، قابل انتقال به بخش مراقبت تحت حاد است.

Title: تاثیر تمرینات هوازی با شدت کم و متوسط بر خودپنداره سالمندان

http://salmandj.uswr.ac.ir/article-1-713-fa.html

اهداف تحقیق حاضر با هدف بررسی تاثیر تمرینات هوازی با شدت کم و متوسط بر خود‌پنداره سالمندان انجام شد.  مواد و روش ها آزمودنی‌های تحقیق شامل 45 سالمند مرد با میانگین سنی 70/2±88/65 سال بودند که به‌طور داوطلبانه انتخاب و به روش تصادفی در دو گروه آزمایش و یک گروه کنترل گمارده شدند. سپس حداکثر ضربان قلب افراد با استفاده از کم کردن سن هر فرد از عدد 220 به دست آمد. در ادامه بر اساس روش تمرینات هوازی با شدت کم (40 تا 50 درصد حداکثر ضربان قلب) و شدت متوسط (60 تا 70 درصد حداکثر ضربان قلب)، حداکثر ضربان قلب هدف در هر یک از آزمودنی‌های گروه‌های تمرینی محاسبه شد. پروتکل تمرینی افراد شامل 8 هفته (هفته ای دو جلسه) تمرین هوازی در قالب آزمون یک مایل پیاده‌روی/دویدن راکپورت بود. شدت تمرین کم تا متوسط نیز با استفاده از بلت‌های مخصوصی که به سینه آزمودنی‌ها متصل شده بود، اندازه‌گیری و کنترل شد. همچنین تمامی آزمودنی‌ها در مراحل پیش آزمون (قبل اعمال مداخله) و پس آزمون (پس از اعمال مداخله)، مقیاس خودپنداره تنسی 2 را تکمیل کردند. یافته ها نتایج مرحله پیش آزمون نشان داد که میانگین نمرات خودپنداره سالمندان مورد مطالعه، اختلاف معنی‌داری با یکدیگر نداشتند (05/0P>). نتایج تحلیل واریانس یک راهه در مرحله پس آزمون نیز نشان داد که گروه تمرین با شدت متوسط دارای خودپنداره بالاتری نسبت به دو گروه دیگر بود (05/0P<). همچنین گروه تمرین با شدت کم دارای نمرات بهتری نسبت به گروه کنترل بود (05/0P<).  نتیجه گیری به‌طور کلی تحقیق حاضر نشان داد که تمرینات هوازی با شدت متوسط، تاثیر مثبتی بر افزایش خودپنداره سالمندان دارد. لذا پیشنهاد می‌شود تمرینات هوازی با شدت متوسط جهت افزایش این ویژگی شخصیتی در سالمندان مورد استفاده قرار گیرد.
